# Venous Thrombosis

**Published:** 1901-11-02

**Authors:** 


### VENOUS THROMBOSIS.

In a recent lecture upon some points connected with thrombosis Sir William Bennett[1] drew special attention to the different courses taken by septic and aseptic thrombi, and especially to the fact that while in the latter embolism is likely to occur during the progress of the thrombotic process, in the case of septic thrombi it is in the later stages while the clot is dissolving away that embolism is to be expected. He related the case of a patient in whom after an operation extensive thrombosis occurred in the left leg. Nearly four weeks after the onset of this she had a sudden attack of difficulty of breathing, from which she nearly died, the result of pulmonary embolism, and later on patches of consolidation occurred in the right lung, showing where the embolus had become lodged. She became very ill, and remained so for a long time, but the point of the case was that this embolism did not happen until three weeks after the formation of the thrombus. "In point of fact, at the time of the occurrence of this embolism the thrombus was commencing to diminish and dissolve away." In contrast with this case he related one of a woman who was admitted with appendicitis, for which an operation was performed. A week later she was going on perfectly well, when it was found, quite accidentally, that one of the lower limbs was swollen. She had not complained of pain, nor was she conscious of anything being wrong, in which this case differed from the other in which pain was a marked symptom. On examination it was found that there was extensive thrombosis in the left lower extremity, which finally involved the femoral and iliac veins. On the day following the discovery of the thrombosis, and whilst the clot was rapidly extending up the thigh towards the groin, a sudden attack of cardiac pain with intense difficulty of breathing occurred, from which she very nearly died. A clot had passed through the heart and had lodged in the lungs giving rise to the usual symptoms. "Here then was a case of thrombosis occurring after an operation in which the patient was doing well, and free from any suspicion of sepsis, followed almost immediately by embolism." In the other case the embolism did not occur until very much later, until, in point of fact, the thrombus had commenced to disappear, whereas in this last case the embolism occurred during the process of the growth of the thrombus. In the first the blood clotted as the result of septic conditions, while in the second the clotting was purely passive, and happened because the vitality of the patient, who was weak and intensely anæmic, had been lowered by the shock of the operation. It is to be remarked that the constitutional symptoms differ greatly in the septic and the aseptic cases. The extent of the thrombosis in this latter (aseptic) case was far greater than in the first (the septic one), yet the first case remained ill, and in fact nearly died, from septic infection, while the aseptic case, directly the clotting ceased to extend, made comparatively rapid progress. The tendency shown by anæmic people and also by many of the "gouty" type to the occurrence of thrombosis when they have to be confined to bed for any length of time must always be borne in mind, and whenever operations of expediency are to be performed upon those who up to the time have been in good health and doing their ordinary work, it is well to keep the patients in bed for some days beforehand so that they may become accustomed to the quiescence before undergoing the operation, or, in other words, may not have to face both risks at the same time. It is not at all uncommon to hear of a man with a broken leg having attacks of "gout." Very often such gout is a purely passive blocking of a vein. It is very obstinate so long as the patient is lying still, but generally rapidly disappears when the circulation is stimulated by movement. In the early stage of thrombosis, of whatever kind, rest is absolutely necessary. Medicinally alkalies with excess of ammonia will do more to hasten the disappearance of these passive clots than anything else.

[1] Clin. Jour., Oct. 16.

